# P-1100. Assessing Hand Hygiene on a Large Scale: Hospital Self-Assessment, Healthcare Workers’ Perception, and HAI Burden in Brazilian Institutions

**DOI:** 10.1093/ofid/ofaf695.1295

**Published:** 2026-01-11

**Authors:** Rafaela M de Moura, André P F de Almeida, Claudia V Silva, Klinger S Faico-Filho, Bruno M Tavares, Brunno C B Cocentino, Ananda Y Z Alvarez, Maira F Moya, Priscila B Garzella, Marianilza L Da Silva, Karen C C D Silva, Thalyta Flores, Ademir J Petenate, Cristiane M R Cristalda, Claudia G De Barros, Sebastian Vernal

**Affiliations:** Hospital Moinhos de Vento, Porto Alegre, Rio Grande do Sul, Brazil; BP – A Beneficência Portuguesa de São Paulo, São Paulo, Sao Paulo, Brazil; Hospital Israelita Albert Einstein, São Paulo, Sao Paulo, Brazil; Universidade Federal de São Paulo, São Paulo, Sao Paulo, Brazil; Hospital Alemão Oswaldo Cruz, São Paulo, Sao Paulo, Brazil; Hospital Sírio-Libanês, São Paulo, Sao Paulo, Brazil; Hospital Moinhos de Vento, Porto Alegre, Rio Grande do Sul, Brazil; BP – A Beneficência Portuguesa de São Paulo, São Paulo, Sao Paulo, Brazil; Hospital Israelita Albert Einstein, São Paulo, Sao Paulo, Brazil; Hcor, São Paulo, Sao Paulo, Brazil; Hospital Alemão Oswaldo Cruz, São Paulo, Sao Paulo, Brazil; Hospital Sírio-Libanês, São Paulo, Sao Paulo, Brazil; Hospital Israelita Albert Einstein, São Paulo, Sao Paulo, Brazil; Ministério da Saúde, Brasília, Distrito Federal, Brazil; Hospital Israelita Albert Einstein, São Paulo, Sao Paulo, Brazil; Hospital Israelita Albert Einstein, São Paulo, Sao Paulo, Brazil

## Abstract

**Background:**

Hand hygiene (HH) is essential for infection prevention. In Brazil, the "Saúde em Nossas Mãos," a nationwide initiative aimed at reducing healthcare-associated infections (HAIs) in intensive care units (ICUs), emphasizes the importance of adhering to the WHO's multimodal strategy to enhance patient safety. We aimed to assess the baseline status of HH in the participating ICUs.

**Table 1:**
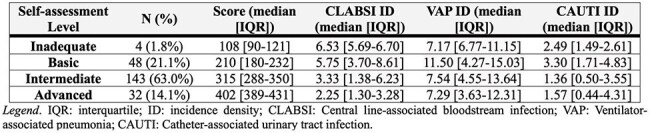
Summary of the self-assessment on hand hygiene: scores and HAI incidence density baseline.

**Table 2:**
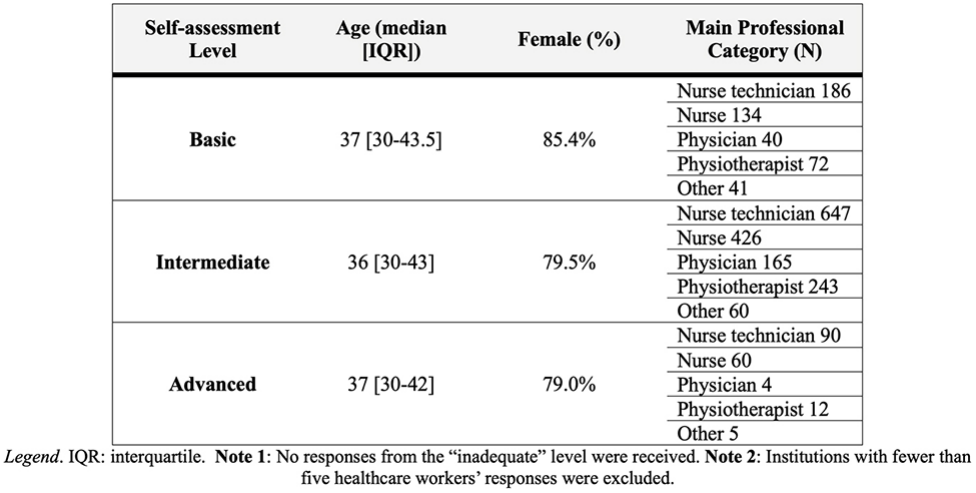
The main features of health workers who responded to the Hand hygiene-HAI perception questionnaire

**Methods:**

The HH Self-Assessment Questionnaire (transculturally adapted by the São Paulo Association of Epidemiology and Control of HAI) was distributed to the Hospital Infection Control Commission of the 293 participating institutions in this quality improvement initiative. The self-report was voluntary and anonymous, reflecting a consensus among the local commission members. Based on the total score, institutions were classified into four levels: Inadequate (0-125), Basic (126-250), Intermediate (251-375), and Advanced (376-500). We also administered a questionnaire on knowledge and perceptions (transculturally adapted by Brazilian Health Regulatory Agency) regarding HH and HAIs among ICU healthcare workers. Additionally, ICUs reported the incidence density for CLABSI, VAP, and CAUTI over a one-year baseline.

**Table 3:**
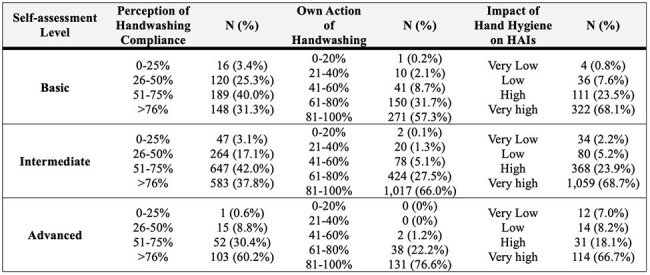
Insights into the HH-HAI perception questionnaire.

**Figure 1:**
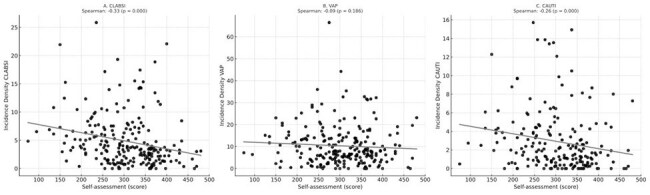
Correlation between self-assessment score and incidence density for each HAI analyzed. A – CLABSI, B – VAP, and C – CAUTI.

**Results:**

A total of 227 institutions submitted the self-assessment (77.4%). Table 1 summarizes the level, scores, and HAI baseline (median, IQR). Figure 1 shows the correlations between the reported score and the incidence density for each HAI analyzed. CLABSI and CAUTI revealed significant negative correlations (R=-0.33, P-value< 0.001; R=-0.26, P-value< 0.001, respectively). 2,297 responses were received from the ICU workers (Basic: 496, Intermediate: 1,604; Advanced: 197). Table 2 summarizes the main characteristics of the professionals. Table 3 includes the frequency of responses related to handwashing compliance perception and own actions on handwashing, and the perceived impact of HH on HAI for each group.

**Conclusion:**

Our findings indicate that Brazilian institutions exhibit varying conditions for HH, still presenting opportunities for improvement. This assessment serves as a baseline for strategic improvements, ensuring progress in HH practices and patient safety aligned with the WHO’s multimodal strategy.

**Disclosures:**

All Authors: No reported disclosures

